# What are the characteristics of ‘sexually ready’ adolescents? Exploring the sexual readiness of youth in urban poor Accra

**DOI:** 10.1186/s12889-015-2620-6

**Published:** 2016-01-05

**Authors:** Adriana A. E. Biney, F. Nii-Amoo Dodoo

**Affiliations:** 1 Regional Institute for Population Studies, University of Ghana, P. O. Box LG96, Legon, Accra Ghana; 2 211 Oswald Tower, The Pennsylvania State University, University Park, PA 16802 USA

**Keywords:** Sexual readiness, Sexual self-concept, Adolescent sexual health, Urban poverty, Ghana

## Abstract

**Background:**

Adolescent sexual activity, especially among the urban poor, remains a challenge. Despite numerous interventions and programs to address the negative consequences arising from early and frequent sexual activity among youth in sub-Saharan Africa, including Ghana, only slight progress has been made. A plausible explanation is that our understanding of what adolescents think about sex and about their own sexuality is poor. In that sense, examining how adolescents in urban poor communities think about their sexual readiness, and identifying characteristics associated with that sexual self-concept dimension, should deepen our understanding of this topical issue.

**Methods:**

A total of 196 male and female adolescents, ages 12 to 19, were surveyed in the 2011 RIPS Urban Health and Poverty Project in Accra, Ghana. The youth responded to three statements which determined their levels of sexual readiness. Other background characteristics were also obtained enabling the assessment of the correlates of their preparedness to engage in sex. The data were analyzed using ordered logistic regression models.

**Results:**

Overall, the majority of respondents did not consider themselves ready for sex. Multivariate analyses indicated that sexual experience, exposure to pornographic movies, gender, ethnicity and household wealth were significantly linked to their readiness for sex.

**Conclusion:**

Sexual readiness is related to sexual activity as well as other characteristics of the adolescents, suggesting the need to consider these factors in the design of programs and interventions to curb early sex. The subject of sexual readiness has to be investigated further to ensure adolescents do not identify with any negative effects of this sexual self-view.

## Background

Adolescent sexual activity is “a fact of life in most African countries, as in many western societies” [1]. However, despite the fact that early sex is a global phenomenon, African nations may be most affected by its consequences [1–3]. Early sexual initiation exposes adolescents to increased risks of STI/HIV infection, unintended pregnancy, and unsafe abortion. In addition, the health of young girls is compromised during childbirth as they are more prone to maternal morbidity and mortality due to their physiological immaturity [4–7]. Infant and child deaths are also likely to occur to adolescent mothers [3]. Furthermore, there is a relationship between young girls’ sexual activity and their school dropout rates [8]. Thus, additional consequences of early sex include female (and sometimes male) adolescents discontinuing school once a pregnancy occurs [5, 9]. Low educational levels reduce the scope of employment opportunities for these adolescents rendering them eligible only for low-income occupations in future [5, 10].

A group of adolescents that requires special attention comprises those living in urban poor communities. This is a vulnerable group, especially susceptible to sexual and reproductive health challenges as a result of the combined effect of poverty and the urban environment [11–14]. In sub-Saharan Africa, studies conducted in urban poor communities in Kenya and Ghana have reported early ages at first sex, multiple sexual partnerships, teenage pregnancy, and youth resolving some of their unintended pregnancies with induced abortions [11, 12, 15, 16]. In Ghana, the proportion of adolescent childbearing slightly increased from 13.3 % in 2008 to 14.2 % in 2014 [6, 17]. The total abortion rate stands at 0.4 abortions per woman, with double the number of abortions occurring to women in the urban settings (0.6 abortions per woman) compared to rural women (0.3 abortions per woman). In addition to this, adolescents are more likely to terminate their pregnancies unsafely [18, 19]. Results from the 2010 Edulink: Urban Health and Poverty study, conducted in urban poor localities in Accra, show that about four out of five women had their first sexual encounter during adolescence compared to 63 % of men. This is comparable to the proportions of women and men in Ghana who stated the same; about 77 % of females and 56 % of males [6, 20]. Young women and men in this community also encountered unintended pregnancies, and half reported inducing or attempting to induce an abortion to resolve that pregnancy. As expected, condom usage among this group was low, with two-thirds reporting non-use the last time they had sex [20]. As the high rate of urbanization continues to city populations in Africa, and the majority of adolescents settle in urban poor communities, these will have implications for the country’s population processes, and economic development. Thus, improving the reproductive health of adolescents in these communities is essential.

To combat the ills of early entry into sexual activity, frequent sexual activity, multiple sexual partnerships, and thereby the risk of induced abortions among adolescents, numerous interventions have been effected in developing countries. Yet, the continuing prevalence of these problems signals the ineffectiveness of these interventions for eliminating these concerns in sub-Saharan Africa. One consideration is that, despite the clear adverse implications for their physical health, educational and occupational chances, and general wellbeing, we simply do not understand why adolescents conduct themselves in the manner they do. Indeed, it is difficult to change people’s behavior without an understanding of the logic that undergirds their actions. This would appear to be more critical when multiple and varied interventions have proven to have limited successes. In order to discern how the beneficiaries of the intended interventions see their sexual selves, which is a useful first step in intervening in their unsafe actions, this study attempts to explore which characteristics of adolescents are related to one dimension of their sexual self-concept, sexual readiness [21–28].

### Sexual self-concept and sexual readiness

The sexual self-concept is “an individual’s view of him- or herself as a sexual person” [25] and has a cyclical relationship with one's sexual intentions, actions, experiences [25], and mental health [29]. Most research on sexual self-concept has been conducted among generally Caucasian, middle income, well-educated, suburban youth in developed countries. Few studies have assessed the sexual self-concepts (SSC) of adolescents in urban poor communities (exceptions to this are [25, 30]) and very few have studied the sexual self-concepts of youth in developing countries (the few exceptions were conducted in Taiwan by [31–33]). Although its methods of measurement vary and can become complex [34], the SSC is considered a useful means to aid in understanding adolescent sexual behavior.

A variety of factors come together to shape young peoples’ sexual self-views [35]. The literature suggests that certain "environmental influences" may be linked to the sexual self-concepts of adolescents and young adults [36]. The influences can emanate from one’s social environment or from significant people [36]. Demographic characteristics, such as gender and age, can lead to differences in sexual self-views. While boys and older adolescents report more sexually adventurous views, their female and younger counterparts exhibit more sexually naïve sexual self-concepts^1^ [22]. Other’s state that males reported less sexual self-efficacious and -esteem views compared to females [26]. Even more studies suggest no such differences between boys and girls, but highlight age differentials [21, 25, 34]. Socio-cultural characteristics such as religion and ethnicity also play a role in shaping their sexual self-concept, through them developing a fear of sexual expression and exhibiting sexual anxiety, respectively [26, 37].

Significant people in adolescents’ lives include their parents or peers, who through conversation communicate their permissive views about sex, this can lead to them forming "arousable" and "agentic" sexual self-concepts that may result in risky sexual behaviors, including initiating sex early [25]. Parents’ demographic characteristics (e.g. their marital status) and socioeconomic characteristics (e.g. their education and income) are linked to adolescents’ sexuality [38]. In the literature, parents with high levels of education and/or income are seen to offer closeness, warmth, monitoring and protection [38, 39]. This has been linked to less sexual activity among adolescents and accordingly suggests less “assertive” and “agentic” sexual self-views. The statement holds true for those residing with their parents, and especially in two-parent homes [38].

This paper is part of a larger study that sought to develop and assess a sexual self-concept scale for adolescents in an urban poor community in Accra [20]. The paper seeks to answer one question: What are the correlates of one critical aspect of adolescent sexual self-concept: “sexual readiness”? Sexual readiness has been linked to motivations to either remain abstinent or initiate sex among adolescents [40, 41]. In these qualitative studies, the subject of readiness emerges from discussions as an intermediary factor on the developmental continuum, providing that entry from abstinence into sexual activity [40]. It is a multifaceted concept consisting of individual characteristics such as the adolescent’s mental, emotional and physical development, age, and social maturity; in essence, it suggests one's preparedness to engage in sex and handle the consequences that may arise. Relationship characteristics that equate one to being ready for sex include being with the right person, which could be either in marriage or in a committed or stable relationship [40–42]. Readiness also has a moral component, according to the adolescents, where the difference between remaining abstinent and initiating sexual activity lies in one’s beliefs about premarital and adolescent sex as being right or wrong. Although sexual readiness may determine whether one starts sex or not [42], sexually experienced adolescents may also view themselves as “not ready”. These research works, typically conducted among African American, low income, urban dwelling boys and girls, provide a sense of what develops their sexual readiness.

Other studies, using survey data, have defined and measured sexual readiness by adopting frameworks and approaches useful for those contexts. While some have assessed adolescents’ stages of sexual readiness and defined them as a function of sexual thoughts, intentions and actions [7, 43], others took into account adolescents’ sexual willingness, autonomy, lack of regret and contraceptive use [28], which is actually a reflection of their sexual competence. The concept of readiness encompasses an array of ideas and renders it a very broad topic that is difficult to define, and these complexities warrant further investigation on the subject. A major weakness in the literature is that sexual readiness has no set definition. In this study, we have identified and incorporated in our measure of readiness key concepts from the existing literature: their preparedness to engage in sex, maturity, and moral views, which are all critical elements that reflect adolescent sexual self-views. These three concepts together provide a more holistic understanding of their perceptions, further validating the measure, especially since solely asking about their readiness to engage in sexual intercourse, is not a comprehensive enough measure [42]. If readiness is that gateway to sexual activity, it connotes an important function preceding their first sex, and if not addressed properly may prolong the harmful consequences of adolescent sex that youth in sub-Saharan Africa continue to experience. In addition, going further to identify characteristics related to their sexual preparedness should lead to ideas on who to target with further research and education, as well as appropriate interventions and programs. Other studies have failed to assess the range of social factors, whether demographic, economic, cultural, or family that may be associated with and ultimately contribute to sexual readiness. Therefore, the study seeks to investigate this as we contribute to the discussion on sexual readiness among a sample of adolescents residing in urban poor Accra. To our knowledge this is the first paper that examines this sexual readiness component of the sexual self-concepts of urban poor adolescents in sub-Saharan Africa.

## Methods

### Data

This paper uses data from the 2011 RIPS Urban Health and Poverty Project.^2^ The main study was conducted in three urban poor localities in Accra (James Town, Ussher Town and Agbogbloshie) surveying men and women in their reproductive ages (15 to 49 years for women and 15 to 59 years for men). Adolescents between ages 12 and 14 residing in sampled households in James Town and Ussher Town were also interviewed as an appendage to the survey. These early adolescents were interviewed with a shortened version of the main questionnaire that focused on their background characteristics, sexual self-concept, and sexual experiences and behaviors.

Sampling of respondents was conducted at two levels: first, 29 enumeration areas (EAs) in the three localities were selected, and from each of these 40 households were systematically sampled. This resulted in a total study sample of 806 households and 1055 individuals (with ages ranging from 12 to 59). However, for this paper, 196 male and female adolescents between ages 12 and 19, residing in James Town and Ussher Town,^3^ are used in our analyses. The majority of respondents residing in this setting affiliate with the Ga ethnic group. These are indigenous residents of the locality, a patrilineal people that were believed to have migrated from Nigeria to Ghana in the 14th century [15]. The towns are by the coast, and thus, the major economic activities practiced include fishing, fish mongering, in addition to trading, and food vending. The second largest ethnic group in this community consists of the Akans, a matrilineal group, who form more than half of Ghana’s population [15].

Ethical clearance to conduct the study was provided by the Noguchi Memorial Institute for Medical Research—Institutional Review Board (NMIMR-IRB) at the University of Ghana, Legon in July 2011. We administered assent or consent forms to adolescents, and further obtained consent from parents or caregivers for adolescents below age 18, prior to the interviews. Our study protocol and practice in the field ensured we provide our respondents’ with privacy during the interview, their information was kept confidential, and small gifts consisting of household items worth about US$3 were distributed after interviews in the entire locality were completed.

### Measurements

The dependent variable is sexual readiness, a composite of the following three items: (1) I think I am ready for sex; (2) I think I am too young to have sex; and (3) I think it is wrong for me to have sex.^4^ These three items were part of a 27 item sexual self-concept inventory, and all 27 items were carefully selected from 13 extant sexual self-concept scales. The initial five-category response for each item, ranging from 1 (strongly disagree) to 5 (strongly agree), was re-coded to create a three-category response indicating 0 (disagree), 1 (neutral) and 2 (agree). The three items loaded heavily (above the value 0.49) on one factor, ensuing exploratory factor analysis, and were summed to create the composite sexual readiness variable which ranges from 0 to 6. The scores indicate that the higher the response, the more the adolescent believes he or she is ready for sex. The ordinal alpha value for the scale was 0.81. For a more detailed process on how the sexual readiness construct was developed see Biney [20]. Descriptive statistics of the latent variable and sexual readiness items are displayed in Tables 1 and 2. Overall, the majority of adolescents were not ready for sex (54.1 % scored ‘0’), while 5.6 % scored ‘6’, indicating a readiness for sex.

**Table 1:** Percentage distribution of sexual readiness items

Items	Frequency	Percent (%)
Ready for sex		
0	153	78.1
1	13	6.6
2	30	15.3
Not young for sex		
0	135	68.9
1	8	4.1
2	53	27.0
Not wrong for sex		
0	137	69.9
1	12	6.1
2	47	24.0
Total	196	100.0

Source: RIPS Urban Health and Poverty Project, 2011

0-disagree; 1-neutral; 2-agree

**Table 2:** Percentage distribution of sexual readiness, background and sexual characteristics of respondents by gender

Characteristics	Total		Female		Male	
	Freq	%	Freq	%	Freq	%
Sexual readiness (Dependent variable)						
0	106	54.1	57	55.9	49	52.1
1	5	2.6	2	2.0	3	3.2
2	37	18.9	20	19.6	17	18.1
3	5	2.6	3	2.9	2	2.1
4	27	13.8	15	14.7	12	12.8
5	5	2.6	3	2.9	2	2.1
6	11	5.6	2	2.0	9	9.6
Socio-demographic Characteristics						
Age						
12–14	68	34.7	38	37.3	30	31.9
15–17	66	33.7	31	30.4	35	37.2
18–19	62	31.6	33	32.4	29	30.9
Highest educational level attained						
Primary and below	65	33.2	36	35.3	29	30.9
Junior High School (JHS)	88	44.9	49	48.0	39	41.5
Senior High School (SHS)	43	21.9	17	16.7	26	27.7
Socio-cultural Characteristics						
Religious service attendance in past month						
At least once a week	113	57.6	56	54.9	57	60.6
At least once a month	39	19.9	23	22.6	16	17.0
Never	44	22.5	23	22.6	21	22.3
Ethnicity						
Akan	43	21.9	23	22.6	20	21.3
Ga/Dangme	131	66.8	65	63.7	66	70.2
Other	22	11.3	14	13.7	8	8.5
Locality						
James Town	78	39.8	41	40.2	37	39.4
Ussher Town	118	60.2	61	59.8	57	60.6
Family Characteristics						
Mother's highest educational level attained						
None/Pre-school	30	15.3	17	16.7	13	13.8
Primary	28	14.3	12	11.8	16	17.0
Secondary	82	41.8	41	40.2	41	43.6
Don't Know	56	28.6	32	31.4	24	25.5
Living arrangements						
Parent/parent & relative	146	74.5	74	72.6	72	76.6
Other relative/non-relative/alone	47	24.0	26	25.5	21	22.3
*Missing*	*3*	*1.5*	*2*	*2.0*	*1*	*1.1*
Parents’ marital status						
Currently married	72	36.7	37	36.3	35	37.2
Not currently married	61	31.1	29	28.4	32	34.0
Never married	22	11.2	13	12.8	9	9.6
Parent(s) dead	39	19.9	22	21.6	17	18.1
*Missing*	*2*	*1.0*	*1*	*1.0*	*1*	*1.1*
Wealth index						
Poorest	39	19.9	17	16.7	22	23.4
Poorer	42	21.4	22	21.6	20	21.3
Middle	39	19.9	18	17.6	21	22.3
Richer	37	18.9	22	21.6	15	16.0
Richest	34	17.4	20	19.6	14	14.9
*Missing*	*5*	*2.6*	*3*	*2.9*	*2*	*2.1*
Sexual communication						
With parents only	101	51.5	54	52.9	47	50.0
With friends only	42	21.4	27	26.5	15	16.0
With both parents and friends	19	9.7	8	7.8	11	11.7
With other(s)	34	17.4	13	12.8	21	22.3
Sexual Characteristics						
Coital experience^a^						
No	156	79.6	75	73.5	81	86.2
Yes	40	20.4	27	26.5	13	13.8
Negative sexual experience						
No	158	80.6	77	75.5	81	86.2
Yes	38	19.4	25	24.5	13	13.8
Exposure to pornographic movies^a^						
No	121	61.7	73	71.6	48	51.1
Yes	73	37.3	28	27.4	45	47.9
*Missing*	*2*	*1.0*	*1*	*1.0*	*1*	*1.1*
Total	196	100.0	102	100.0	94	100.0

Source: RIPS Urban Health and Poverty Project, 2011

^a^Significantly different by gender at *p* < 0.05

The sexual characteristics of respondents include their coital experiences (whether they ever had sexual intercourse with a member of the opposite sex) and negative sexual encounters (whether they were forced to have sex or forced to engage in a sex act but not have sexual intercourse). In addition, their exposure to pornographic movies denotes whether the respondent ever watched a pornographic movie or not.

Socio-demographic characteristics include the age, gender and highest educational level of the respondent. Socio-cultural variables comprise of their religiosity, measured by number of religious services attended in a month, the locality the respondent resides in, and his/her ethnicity, which was categorized as Ga/Dangme, Akan, and Other. Significant people influences were measured by family characteristics which include the respondent’s mother’s highest educational level, his/her current living arrangement, parents’ current marital status, the sexual communication (or lack thereof) that takes place in the household, and the household wealth. This wealth measure was computed using assets owned by each household (for example, a radio, television, car, mobile phone, fishing net, etc…), in addition to the materials used to construct the homes they dwelled in. The items underwent principle components analysis (PCA) and a wealth index was created using the first factor. The index was then grouped into quintiles, and the lowest 20 % in the index were labeled as the poorest respondents, the poorer respondents were the next lowest 20 %, this ran through to the richest being the top 20 % in the index.

### Analyses techniques

Descriptive statistics were used to assess the characteristics of respondents using percentages along with cross-tabulations by gender. Pearson chi-square tests were employed to indicate the factors that differed significantly with gender. Ordinal (or ordered) logistic regression analysis was the main statistical technique used since the dependent variable was an ordered scale ranging from 0 (not sexually ready) to 6 (very sexually ready). We examined associations between the socio-demographic, socio-cultural, family, and sexual characteristics of adolescents, and the sexual readiness dimension of their sexual self-concept. Results are displayed as odds ratios. The statistical software package STATA was employed to undertake the analyses.

## Results

### Participants’ characteristics

A total of 196 youth, ages 12–19 years, residing in James Town and Ussher Town, participated in the study. The background characteristics of the adolescents are shown in Table 2, and include gender differences which aids in simple comparisons of their characteristics by gender. The majority of the respondents were students (73.4 %) and they were either in (or had completed) junior high school (JHS). Gender differences reveal that more boys than girls were still in school. Also, a higher proportion of males compared to females had completed—or were currently in—senior high school. About four-fifths of the respondents were Christians; and the majority reported being ‘religious’, that is, they attended a religious service at least once a week in the month preceding the survey (57.6 %). They mostly resided in Ussher Town (60.6 %) and were from the Ga/Dangme ethnic group.

As indicated in Table 2, the majority of respondents had mothers who had completed secondary school. Just over a quarter of respondents did not know the highest educational levels attained by their mothers. More respondents stated that their parents were currently married (36.7 %), while a similar proportion (31.1 %) stated they were currently not married. Most of the respondents lived with their parent(s) and more households were categorized into the poorer wealth quintile. In relation to sexual discussions with parents, more than half of the adolescents talked to their parents when they had sexually related questions or issues. Gender patterns were similar across the family characteristics, except that most female respondents did not know their mothers’ educational levels compared to the males. Also, a higher proportion of boys were reported to reside in the poorest households than girls (23.4 vs 16.7 %). Finally, a smaller percentage of boys cited discussing their sexual concerns with friends only compared to girls (16.0 vs 26.5 %).

The sexual characteristics of adolescents indicate that while one-fifth of them had experienced sexual intercourse, a similar proportion stated that they had ever been forced to engage in a sex act (not necessarily sexual intercourse) or to have sex. Of the 19.4 % who stated this occurring, about 58 % had been forced to engage in a sex act only, 15.8 % had been raped only, and 26.3 % had experienced both negative sexual encounters (see Table 3). A higher percentage of females stated this (24.5 vs 13.8 %). Also, while close to one-half of the boys had ever watched a pornographic movie, only just over a quarter of the girls stated the same (Table 2). Their engagement in sexual intercourse and their exposure to pornography were the only characteristics significantly different by gender.

**Table 3:** Percentage distribution of adolescents who ever encountered a negative sexual experience; were forced to have sex, engage in a sex act or both

Characteristic	All		Girls		Boys	
	Freq	%	Freq	%	Freq	%
Negative sexual experience						
No	158	80.6	77	75.5	81	86.2
Yes	38	19.4	25	24.5	13	13.8
Total	196	100.0	102	100.0	94	100.0
Negative sexual experience categories						
Forced sex only	6	15.8	5	20.0	1	7.7
Forced into sex act only	22	57.9	12	48.0	10	76.9
Forced into both	10	26.3	8	32.0	2	15.4
Total	38	100.0	25	100.0	13	100.00

Source: RIPS Urban Health and Poverty Project, 2011

In summary, the backgrounds of the respondents indicate that they are educated, “religious”, Ga-Dangme adolescents. They reside with one or both parents in homes categorized as poorer on the wealth index. One out of every five adolescents had ever had sex or ever been coerced into sex or a sexual act. Results also suggest that in this community, females are sexually experienced at earlier ages than males, while males have more exposure to sexually explicit movies. These outcomes speak to the vulnerability of young women in the community who are experiencing higher rates of sexual coercion and risky behavior than their male counterparts.^5^

### Multivariate analyses

Ordered logistic regression results are displayed in Table 4. Two models were conducted; the first solely incorporated the three sexual variables and the second included the background variables. Although regression models may be used to predict and establish causality, this feature was not important to the study. Rather, multivariate analyses were conducted to determine correlates of sexual readiness upon testing for associations. Results from Model 1 show that sexually active respondents see themselves as significantly more sexually ready than their abstinent counterparts. In addition, adolescents with exposure to pornographic movies were more ready than those with none. After including their background characteristics, the outcomes still hold for coital experience and pornographic exposure, where these respondents were also significantly more ready for sex than their peers.

**Table 4:** Ordered regression models displaying sexual, socio-demographic, −cultural and family correlates of adolescents’ sexual readiness

	Model 1		Model 2	
Characteristic	Odds Ratio	95 % Confidence Interval	Odds Ratio	95 % Confidence Interval
Coital experience				
No (RC)	1.000		1.000	
Yes	9.339***	4.18–20.88	19.585***	6.85–56.03
Negative sexual experience				
No (RC)	1.000		1.000	
Yes	1.014	0.47–2.17	1.362	0.58–3.19
Exposure to pornography				
No (RC)	1.000		1.000	
Yes	2.043*	1.12–3.73	2.355*	1.07–5.19
Gender				
Male (RC)			1.000	
Female			0.420*	0.20–0.87
Age group				
12–14 (RC)			1.000	
15–17			1.031	0.39–2.74
18–19			0.825	0.26–2.56
Highest educational level attained				
Primary and below (RC)			1.000	
JHS			0.790	0.33–1.90
SHS			0.493	0.16–1.52
Religious service attendance in past month				
At least once a week (RC)			1.000	
At least once a month			0.914	0.38–2.19
Never			1.301	0.56–3.01
Ethnicity				
Akan (RC)			1.000	
Ga/Dangme			3.427*	1.29–9.07
Other			4.212*	1.11–16.04
Locality				
James Town (RC)			1.000	
Ussher Town			1.776	0.86–3.651
Mother's highest educational level attained				
None/Pre-school (RC)			1.000	
Primary			2.276	0.65–7.99
Secondary			1.805	0.65–5.02
Don't know			0.961	0.32–2.84
Living arrangements				
Parent(s)/parent & relative (RC)			1.000	
Other relative/non-relative/alone			1.251	0.57–2.76
Household wealth quintile				
Poorest (RC)			1.000	
Poorer			3.574*	1.27–10.06
Middle			1.905	0.65–5.61
Richer			0.996	0.29–3.39
Richest			0.821	0.24–2.78
Parents' marital status				
Currently married (RC)			1.000	
Not currently married			0.452+	0.18–1.12
Never married			0.670	0.21–2.11
Parent(s) dead			1.261	0.46–3.44
Sexual Communication				
With parent(s) only (RC)			1.000	
With friend only			0.433+	0.18–1.06
With both parents and friends			0.645	0.18–2.29
With other(s)			2.268+	0.90–5.71
/cut1	0.859	0.45-1.26	2.422	0.54-4.30
/cut2	0.990	0.58-1.40	2.601	0.71-4.49
/cut3	2.143	1.63-2.66	4.030	2.06-6.00
/cut4	2.342	1.81-2.88	4.293	2.32-6.27
/cut5	3.698	2.98-4.42	5.770	3.72-7.82
/cut6	4.151	3.35-4.95	6.276	4.18-8.37

Source: RIPS Urban Health and Poverty Project, 2011

***p < 0.001; **p < 0.01; *p < 0.05; +p < 0.10; RC = Reference Category

Model 1: Pseudo R^2^ = 0.1063, *n* = 194; Model 2: Pseudo R^2^ = 0.2085, *n* = 184

Proportionality of odds test: Model 1: p > chi2 = 0.9474; Model 2: p > chi2 = 0. 4705

Three other factors were associated with their sexual readiness. Gender was significantly related to one’s sexual self-view, where girls were less ready for sex than boys. Ethnicity was also a significant correlate of sexual readiness. The matrilineal Akan ethnic group, found across middle and southern Ghana, was significantly less ready compared to the patrilineal Ga/Dangmes and Other ethnic groups (consisting mostly of the Guans, Ewes and Mole-Dagbanis). In addition, respondents in the poorer household wealth quintile were more sexually ready than their poorest colleagues. This suggests that the poorest youth in this community are not as ready for sex when compared to the poorer ones.

Furthermore, as shown in Table 5, the predicted probability of an adolescent falling into a particular sexual readiness category was assessed following regression analysis. The selected respondents were those with characteristics that indicated a significantly higher degree of sexual readiness; specifically, sexually active, pornography viewing, poorer, non-Akan males. The probability of these adolescents falling into the highest category of ‘6’ on the readiness scale was high at 0.8724, further indicating, to an extent, that the identified group exhibits an extreme amount of sexual readiness.

**Table 5:** Distribution of predicted probabilities of adolescents at their various degrees of sexual readiness for significant variables in the model

Sexual Readiness Categories	‘Sexually active’ only	‘Exposed to pornography’ only	‘Males’ only	‘Ga/Dangme’ only	‘Other ethnicity’ only	‘Poorer wealth’ only	All significant characteristics
0	0.1074	0.4246	0.4449	0.4547	0.2583	0.3180	0.0031
1	0.0185	0.0444	0.0447	0.0448	0.0359	0.0402	0.0006
2	0.2495	0.3176	0.3105	0.3069	0.3407	0.3414	0.0116
3	0.0635	0.0409	0.0389	0.0379	0.0587	0.0523	0.0045
4	0.3351	0.1271	0.1190	0.1153	0.2147	0.1780	0.0613
5	0.0763	0.0175	0.0162	0.0156	0.0343	0.0266	0.0465
6	0.1498	0.0279	0.0258	0.0248	0.0574	0.0435	0.8724

Source: RIPS Urban Health and Poverty Project, 2011

## Discussion

This study sought to identify factors that were significantly associated with an adolescent’s sexual readiness. In this context, sexual readiness brought together concepts of being ready, being right and not being too young to have sex, indicating that views about preparedness, morality, and maturity are key components used to assess the readiness dimension of their sexual self-views. These are also individual characteristics that youth in an inner city US based study mentioned as “defining” readiness for sex [40]. More specifically, the question that explicitly asks their views on how ready they are for sex gives clear indication of how “set” they are to initiate sex [27]. However, results show that not too many adolescents in the study community identified with this sexual self-view as more than half reported no readiness whatsoever. An element of culture and societal norms could be driving this perception since there is some societal disapproval and shame associated with premarital sex in most cultures [39]. But, we do not consider this to fully explain these views since Ga Mashie is known to be a community where sexual activity among young people is not harshly criticized. It is subtly encouraged, especially among girls, as a survival strategy, which is typical in most urban poor settings [11, 15]. This result needs further examination.

Our findings show that sexual experience—whether through media exposure or from practical experience—denotes greater readiness. The result that coital experience is a predictor of sexual preparedness is similar to those found in the sexual self-concept literature. These studies highlight adolescent and young adults’ sexual experiences as being significantly linked to sexual self-concepts that denote sexual assertiveness, agency, arousability, adventure and drive [22, 23, 25]. Since it was not an aim to establish causality, the issue of time sequencing was not considered in assessing the correlates. However, the concept of sexual readiness and coital experience most likely relate bi-directionally. Thus, one’s readiness views could bring about initiation into sex and having had sex could provide that feeling of readiness. The influence of sexually explicit media on adolescents' sexual self-views also emerged. In her longitudinal study, Aubrey [40], discovered that among young women, the number of hours of TV watched a day, watching primetime dramas and watching soap operas were linked to a worsening of their sexual self-concept [44]. Although her results speak to the influence of less sexually explicit forms of media, it does give insight into the role of the media in portraying sexual scripts that educate adolescents on how to behave in these situations. This instruction could leave them “better equipped” to engage in sexual behavior, with the level of risk they are engaging in resembling what they have been exposed to. Again, the inverse relationship could exist where exposure may result in a feeling of readiness or pre-existing readiness could foster the impulse to view pornographic material. In addition, those who faced negative encounters with sex were not more ready than their peers, which could suggest that their forced exposure to sexual acts and/or sexual intercourse had no effect on perceptions about their readiness.

Adolescent sexual activity (and pregnancy), according to the rational adaptation theory [45], is a means through which adolescents derive benefits. It provides an escape from poverty, and gets them into stable relationships, gives them a home and a family for those who lack parental monitoring and control [1]. Finding that the poorer adolescents had these mindsets was expected; however, it is surprising that the poorest adolescents were not as ready as their less poor counterparts. This outcome implies that respondents who are just above being the most worse off in their community may behave differently from those in the other groups by indication of their sexually ready attitudes. Thus, in targeting youth with programs to improve their reproductive health, this group should be addressed differently from the others. However, to fully ascertain this, other variables that can best measure the adolescent’s household’s wealth and socio-economic status must be used. Another outcome that needs further clarification is this relationship between ethnicity and sexual readiness. The finding that Akans are the ethnic group least ready for sex raises questions about assimilation and acculturation of the Akans into an indigenous Ga society. The localities under study consist mostly of respondents with Ga ethnic heritage. In the confines of Accra, this group is seen as a people with a pro-natalist affinity, thus the societal norm would be for them to start relationships and childbearing earlier [15]. Apart from their lineage differences, certain customs and traditions also differ. For example, the Ga tend to practice a duolocal residential system among married people, where husbands and wives may still reside in their family homes [15]. This and other cultural differences could result in socialization that renders some adolescents more sexually ready than others. The Akans and other ethnic groups can be considered migrants, although some may have settled for years in Accra. It could be that Akan adolescents may not have ascribed to the same ideals as their Ga counterparts, while the Others consisting of smaller ethnic groups may have. In order to test this idea further, variables related to migration such as duration of stay in the localities as well as parents’ migration status and attitudes in relation to sexual readiness need to be examined.

Knowing that males consider themselves more sexually ready than females speaks to the gender differentials in the community, which are similar to global views [7, 27]. Butler et al. [37] noticed that among a sample of mostly African American preadolescent boys and girls, larger proportions of boys progressed across the stages of sexual readiness after six months while more girls remained stable or retrogressed. In that setting boys initiated sex earlier than girls; however, this is not the case for youth in the localities under study (as well as across Ghana) where generally higher proportions of girls start sex prematurely compared to boys. Despite the later sexual debut of boys in our sample, they still deemed themselves to be more sexually ready than girls. Nonetheless this outcome was expected since societal norms indicate that girls are raised to think about sex negatively as well as be sexually passive and indifferent [23, 46, 47]. For boys, on the other hand, the sexual is “central to the validation of their masculinity” [23].

Various dimensions of the sexual self-concept, including sexual readiness, are thought to be predicated on one’s age [7, 22, 27]. The surprising finding that age was not significantly associated with their preparedness to engage in sex indicates similar states of sexual readiness among this sample of early (12 to 14 year olds), middle (15 to 17 year olds) and late (18 to 19 year olds) adolescents. Indeed, key unobserved characteristics may circumvent age to influence their sexual self-views. A few that we consider as important for this context are parental (or familial) attitudes, and financial and physical presence variables such as, their permissive views about sex, financial support, monitoring, etc… may suggest "normative standards" for the youth to follow [48]. These factors could be masking the effect of age and may ultimately play a role in shaping their sexual self-views [39]. With this being the case, the issue of their social maturity, defined in this context as being prepared for adult experiences and consequences, suggests another important predictor of their sexual self-views other than age. Despite age, adolescents may have reached certain milestones that could warrant them the right to deem themselves sexually ready in their societies.

The strengths of the study are many. We derived a new ordinal measure of sexual readiness, that incorporated a new means of assessing the readiness sexual self-view of both virgins and non-virgins. Our study also went further to relate their readiness to adolescents’ characteristics using multivariate analysis, and this provided the chance to control for the effects of other characteristics in our models. We find that net of the other factors, sexual experience, exposure to pornography, gender, ethnicity, and wealth were related to their self-views. Apart from statistically significant gender differences among pre-teens’ readiness to learn about sex and engage in sex, no other studies report the findings that we do. Due to these gender differences which seem to be almost universal, they must be considered as important when considering the needed development programs targeted at adolescents’ sexual readiness. However, additional studies are needed in order to state further comparisons between our findings and those conducted in the US and UK on sexual readiness. Despite this, our study advances the literature on sexual readiness through assessing this new measure appropriate for the Ghanaian context and identifying which characteristics of urban poor adolescents are ascribed to a particular readiness self-concept.

This study is not without its limitations. The small sample size limited any additional analyses to explore the correlates separately by age or gender, which could have shed more light on the factors associated with adolescents’ sexual readiness. Therefore, extensions to this study include administering the sexual readiness scale to a larger sample as well as to youth in similar urban poor localities in Accra to broaden our understanding of this dimension. However, our limited sample size does not discount this study’s contribution to the literature. It provided exploration of an arena that we know little about. Given that it is just as possible to commit a type I error in a small sample as in a large one, the fact that we find something interesting in our results warrants the need for further work in this area. Nevertheless, we recognize that we cannot draw any definitive conclusions from our results. Overall, the results helped, to an extent, explore the ‘who is ready for sex’ question; however, further research through qualitative means is needed to understand why adolescents with these characteristics perceive their readiness for sex. In addition, longitudinal data, through panel studies, could provide the appropriate time sequencing to aid inference of causality, further ascertaining the relationships between the correlates and their preparedness for sex.

## Conclusion

Although the majority of adolescents residing in this urban poor community in Accra report not being sexually ready, characteristics of those that are, include them being sexually experienced and having some exposure to sexually explicit material. Other correlates of their sexual readiness include being male, non-Akan and in the poorer household wealth quintile. Since these adolescents vary in their degree of sexual readiness, any programs or interventions developed to improve their sexual and reproductive health by targeting the sexual readiness dimension must be tailored according to adolescents’ diverse characteristics. It is imperative that gender differences in sexual readiness are emphasized since they are relevant in varied settings, with boys exhibiting higher levels of readiness than girls. It also behooves us to further explore this issue of young people’s sexual readiness, which will extend the literature on protective and risk factors of adolescents sexual behavior, and the eventual development of appropriate programs for at-risk adolescents.

## Footnotes

1 Buzwell and Rosenthal [22] determined five clusters representing five distinct sexual self-concept dimensions/styles of adolescents. They were categorized into sexually naïve, sexually unassured, sexually adventurous, sexually competent and sexually driven groups. They styles were a reflection of their responses on the sexual self-esteem, sexual self-efficacy and sexual attitude scales. For example, the sexually naïve adolescents exhibited low sexual self-esteem, low sexual self-efficacy, and high sexual commitment and anxiety attitudes, while the sexually adventurous youth exhibited the opposite.

2 These data are from the second of three waves of a project conducted by the Regional Institute for Population Studies (RIPS), on urban poverty, population and health issues. Two of the localities (James Town and Ussher Town) are considered urban poor settings with indigenous Ga residents, while Agbogbloshie is more of an informal (slum) settlement consisting of more transient dwellers.

3 Adolescents between ages 12 and 14 were not interviewed in Agbogbloshie, hence the 15 to 19 year olds that were interviewed were also not included in this sample.

4 Items 2 and 3 were reverse coded into ‘I do not think I am too young to have sex’ and ‘I do not think it is wrong for me to have sex’. This was done to suggest that agreement meant adolescents “owned” their sexuality.

5 Further analyses show that a significantly higher proportion of girls state their first sexual encounter was with a boyfriend who was older than them. In addition, with the exception of males engaging in sex with more casual partners, females were more likely to exhibit risky sexual behavior which was largely characterized by their non- or inconsistent use of condoms with sexual partners.
